# Nutritional needs and health outcomes of ageing cats and dogs: is it time for updated nutrient guidelines?

**DOI:** 10.1093/af/vfae008

**Published:** 2024-06-20

**Authors:** Emma N Bermingham, Keely A Patterson, Anna K Shoveller, Karl Fraser, Christina F Butowski, David G Thomas

**Affiliations:** AgResearch, Palmerston North, New Zealand; AgResearch, Palmerston North, New Zealand; Massey University, Palmerston North, New Zealand; University of Guelph, Canada; AgResearch, Palmerston North, New Zealand; Riddet Institute, Palmerston North, New Zealand; AgResearch, Palmerston North, New Zealand; Massey University, Palmerston North, New Zealand

**Keywords:** cognitive function, healthspan, inflammageing, lifespan, nutrients, sarcopenia, senior

**Implications**

- While cats are classed as senior at 10 years of chronological age, physiological and health changes occur from 8 years of age and it appears that diet may influence the ageing process.
- Dogs are classed as senior at 12 years for smaller dogs and 10 years for larger breeds. Due to differences in longevity associated with breed size a definite age that dogs start to experience changes is difficult to establish.
- Despite our pets ageing, living in extreme cases to 30 + years, there are no explicit nutritional guidelines for feeding ageing animals. Increased scientific knowledge around the specific nutritional requirements of ageing cats and dogs is required.
- Many of the underlying physiological processes (e.g., immune function) and age-associated health conditions (e.g., cognitive decline) respond to nutritional intervention. This suggests that nutritional and regulatory guidelines, should consider recommendations for ‘senior+’ pets.
- Due to the unique nutritional requirements of cats and dogs, more specific knowledge around the mechanisms of ageing is required.

## Introduction

In October 2023, Bobi, the oldest dog in the world, died. His age was estimated to be 31 years and 165 days. Creme Puff, the oldest cat in the world, who, in August 2005, passed away at the age of 38 years and 3 days. While certainly these two pets were at the extreme end of lifespan, it is undeniable that our pet cats and dogs are living longer lives; this has been attributed to improved veterinary care and diet formulation (Bellows et al., 2015, 2016). Indeed, it is currently estimated that 20% to 40% of pets are “old” being greater than 11 years of age (Bellows et al., 2015, 2016). However, information relating to how our pets age, and how we can improve not only their lifespan (i.e., chronological age), but healthspan (i.e., longer, *healthier* lifespan) is lacking. Therefore, the aim of this review is to summarize recent publications and current global initiatives investigating ageing and its processes in cats and dogs. Further, we aim to review the nutritional requirements of our ageing cats and dogs, including evidence for specific nutrients which have health benefits for common ailments associated with ageing.

## Nutritional requirements of cats and dogs

There are several differences between the nutrient requirements of cats and dogs; these include specific amino acids (e.g., taurine is an essential amino acid for the cat, but not the dog), fatty acids, vitamins, and minerals. Additionally, there are differences in the minimum and maximum inclusion levels of these nutrients. Evolution of the two species is likely the cause of these differences, supporting the notion that cats are obligate carnivores (requiring animal-tissues to survive), whereas dogs are facultative carnivores—requiring these animal proteins and fats for optimal nutrition, but being able to survive via scavenging a range of food resources (Plantinga et al., 2011; Bosch et al., 2015). The nutrient requirements of cats and dogs are vastly different to those of their owners, and reflect an evolutionary adaptation to a diet with high levels of animal tissue. Therefore, cats are not small dogs, and nor are cats and dogs, small humans.

Despite the pet food industry existing since the 1950s, there is relatively little scientific literature pertaining to the specific nutrient requirements of cats and dogs; including energy requirements which underpin feeding guidelines and nutrient availability. The consequence of our sparse nutritional knowledge of the cat and dog means that when it comes to interpreting the requirements of the ageing cat and dog from experimental studies, we are heavily reliant on translating such findings from omnivores.

The global pet food industry generally adheres to two nutritional regulatory guidelines; namely the American Association of Feed Control Officials (AAFCO) and the European equivalent, European Pet food Industry Federation (FEDIAF). Both regulatory bodies update their guidelines frequently, and while they may make changes based on new information, they are largely based on data outlined by the “Nutrient Requirements of Dogs and Cats”, last updated by the National Research Council (NRC) in 2006 (National Research Council, 2006) and therefore do not include data published since then. In addition, many of the nutritional requirements stipulated by AAFCO and FEDIAF are based on extruded-kibble formulations which are recognized as being less digestible than other formats (e.g., retorted or raw diets), or on semi-purified diets which increase the nutrient bioavailability and don’t represent a typical pet food. This is increasingly important with the rapid shifts in pet food manufacturing, product formulations (i.e., extruded kibble, retorted can diets, air/freeze-dried, sous vide, etc.), and ingredients used.

## How does health change in ageing cats and dogs?

Much of the scientific literature around ageing in the cat and dog stipulate that ageing is not a disease. Indeed, ageing has been defined as a natural ‘series of life stages’, whereas senescence is the deterioration of the health and quality of life (Case et al., 2011). The definition of life stages has typically followed a chronological ordering based on years of life, especially for the cat (Quimby et al., 2021). Typically, cats have been classed as ‘senior’ when they are older than 8 to 10 years of age (Ray et al., 2021). For the dog, body size and breed have a major influence on life stage, with larger dogs reaching ‘old age’ at an earlier chronological age than smaller dogs (Creevy et al., 2019). However, more recent work has re-classified life stages of the cat and dog based on diagnosis of disease (Salt et al., 2023), and suggested additional age classifications on this basis (Table 1).

**Table 1.** Age classifications of cats and dogs with additional age classifications as suggested by Salt et al. (2023), indicated by an asterix

Cat		Dog		
Kitten	<1 year		Puppy	<1 year
Young/youth	1-4 years	Toy	Youth*	1-6 years
			Midlife*	7-11 years
			Senior	12-13 years
			Super-Senior*	≥ 14 years
Early midlife*	5-9 years	Small	Youth	1-6 years
			Midlife	7-11 years
			Senior	12-13 years
			Super-Senior	≥ 14 years
Late midlife*	10-11 years	Medium	Youth	1-5 years
			Midlife	6-9 years
			Senior	10-13 years
			Super-Senior	≥ 14 years
Senior	12-13 years	Large	Youth	1-5 years
			Midlife	6-9 years
			Senior	10-11 years
			Super-Senior	≥ 12 years
Super-senior*	≥ 14 years			

### Physiological decline in ageing cats and dogs

**Figure 1.**
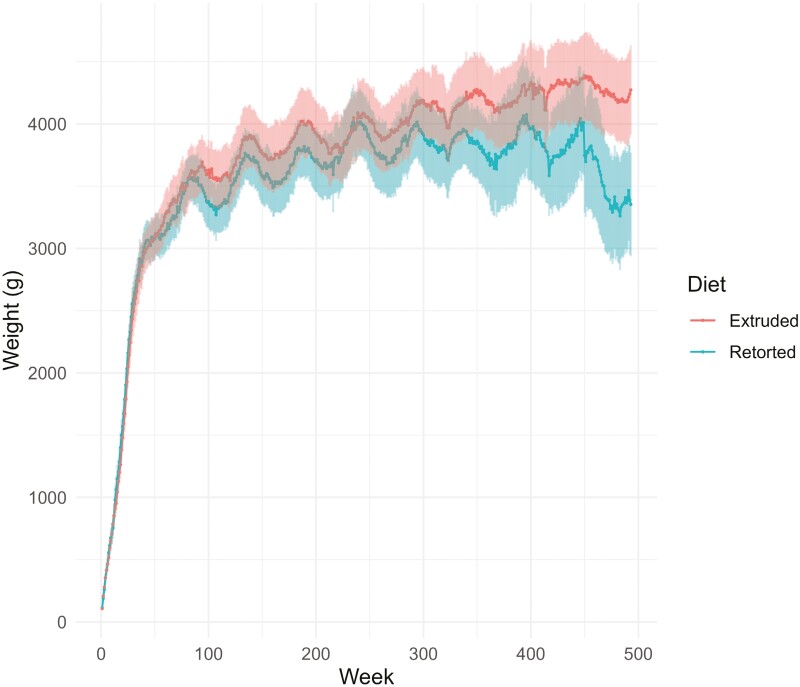
Weekly bodyweight in cats fed either an extruded kibble (*n* = 10) or retorted canned (*n* = 10) diets for 12 years. From this graph, we can see a) that there is a natural rhythm associated with bodyweight across a year; typically increasing in the months leading up to winter and decreasing in spring and b) there is very little difference between the bodyweights of cats fed either diet until approximately 8 years of age where the cats fed the extruded diet maintained their ‘winter weight’.

**Figure 2.**
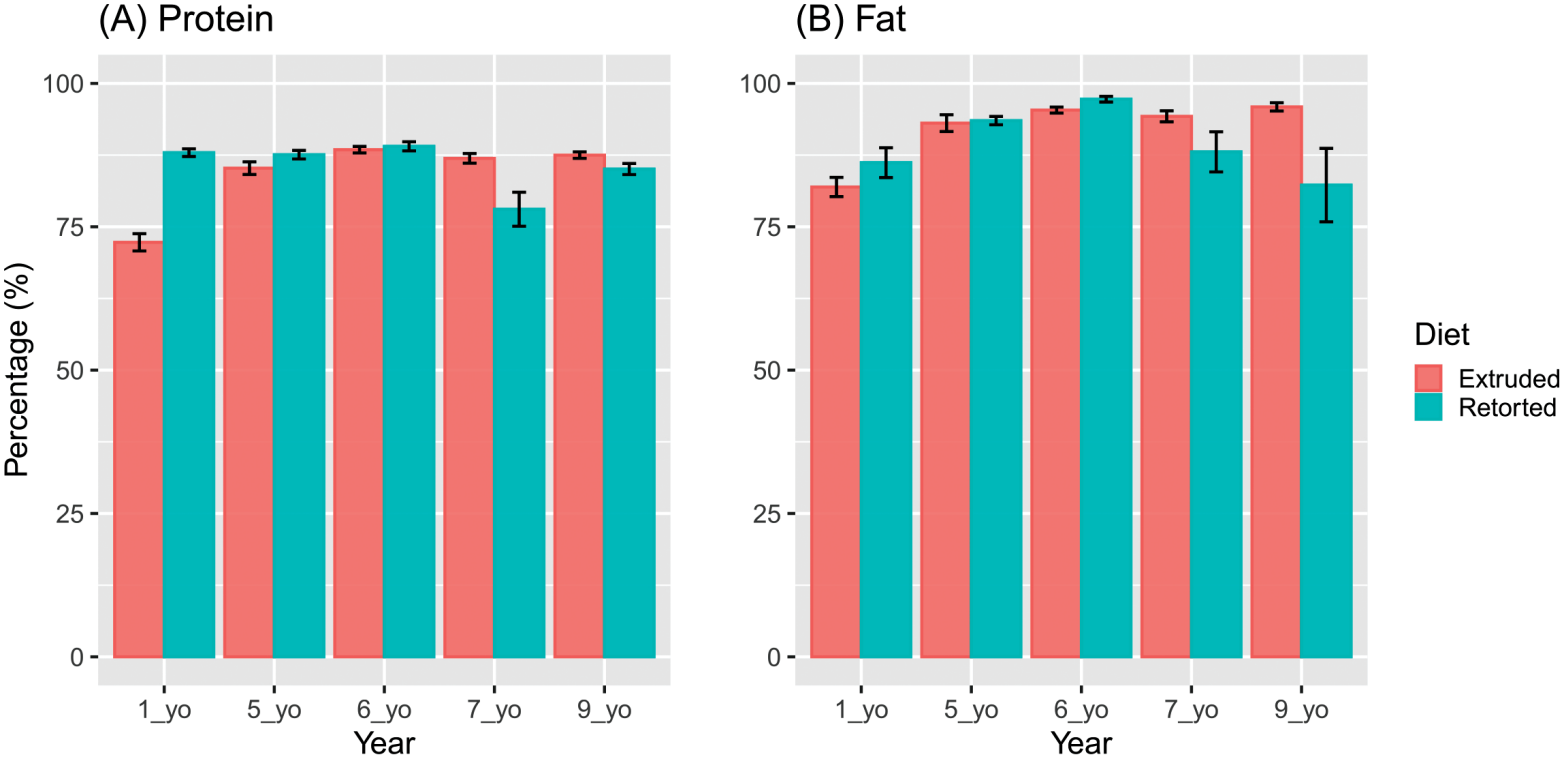
Apparent total tract digestibility of crude protein and fat in the domestic cat at 1, 5, 6, 7, and 9 years of age (yo) (from Bermingham et al 2018 and unpublished results).These results show that a) protein digestibility in young cats fluctuates with age and b) fat digestibility is affected by age, but there is no difference in cats fed retorted or extruded diets.

While it is understood that reduced ability of cats to digest nutrients and utilize energy stores may begin after 7 years of age (Bellows et al., 2015, 2016), more recent research suggests dietary format influences this. For example, cats fed ad libitum extruded or retorted diets were able to maintain a healthy weight range until approximately 8 years of age, at which point bodyweight declined for animals consuming the retorted diet (Figure 1). Research indicates that fat digestibility and to a lesser extent protein digestibility (Harper, 1998; Perez-Camargo, 2004; Teshima et al., 2010; Bermingham et al., 2013, 2018), decrease with age in the cat; however, the extent to which they are affected may be dependent on dietary format and nutrient content (Figure 2). Changes in nutrient digestibility in the ageing cat may be due to changes in intestinal morphology (Peachey et al., 2000), as there are limited impacts of ageing on intestinal transit time and gastric emptying time between young and senior/super-senior cats (Papasouliotis et al., 1998; Peachey et al., 2000). This suggests that there is a defined window in which the cat is likely to be undergoing significant changes in its metabolism and physiology which may present an opportunity for nutritional interventions.

Research suggests that dogs experience no change in intestinal permeability or absorptive capacity as they mature from adult to seniors and beyond, however changes in intestinal morphology have been documented (Garden et al., 1997; Weber et al., 2002; Kuzmuk et al., 2005). The impacts of age on nutrient digestibility are affected by both dietary format and ingredient composition and warrant further investigation (Sheffy et al., 1985; Taylor et al., 1995; Larsen and Farcas, 2014). For example, observations suggest that increasing the amount of total dietary fiber appears to mitigate the age-associated impacts on fat digestibility (Schauf et al., 2021). Additionally, it is often reported that ageing dogs require 20% to 30% more dietary protein to maintain muscle mass (Laflamme, 2005).

### What is the role of the gut microbiome in ageing?

**Figure 3.**
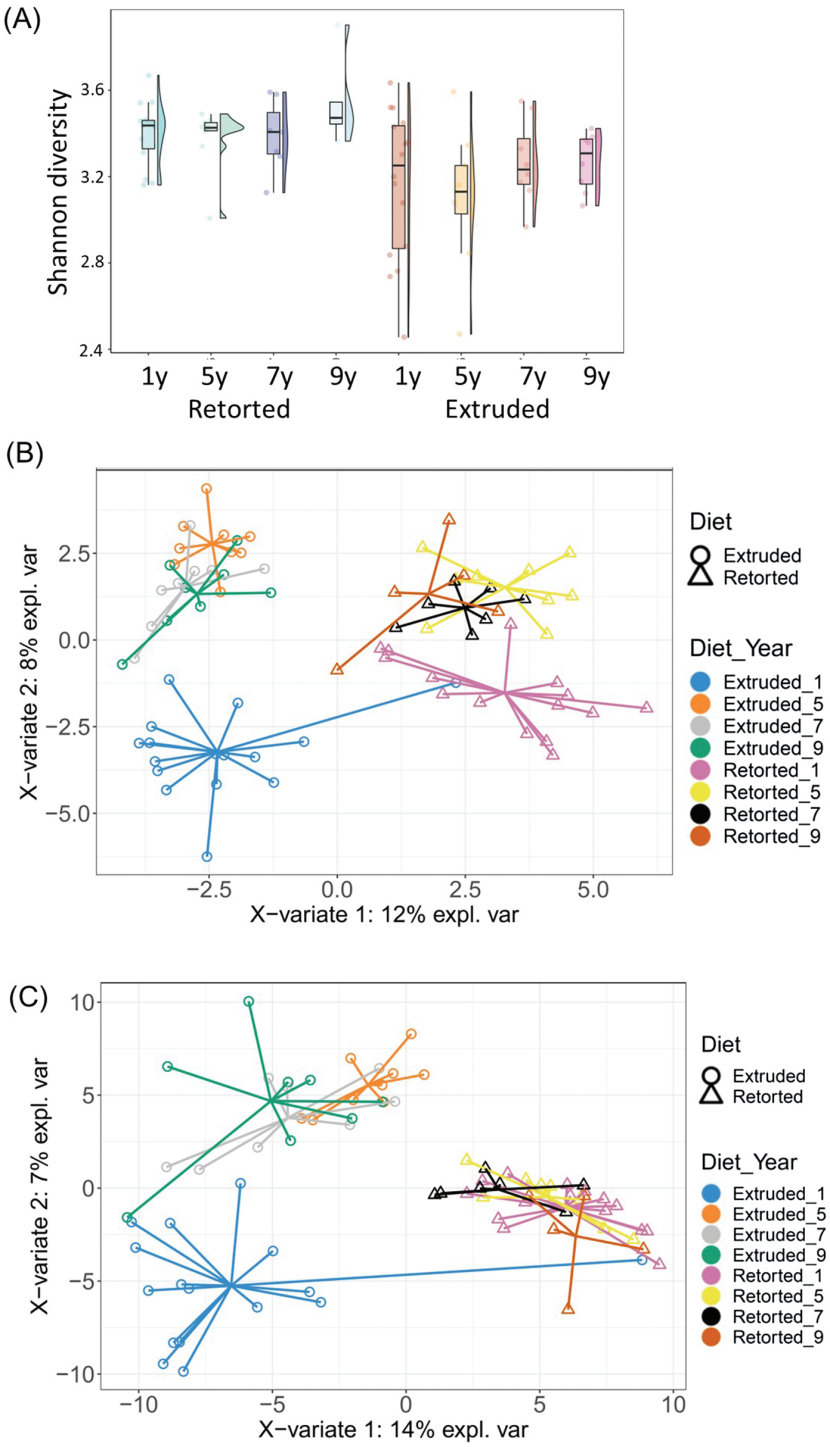
Shannon diversity indices (A) of cats (*n* = 16) fed either a retorted or extruded kibble for 1, 5, 7, and 9 years. Partial least squares discriminant analysis (PLSDA) of taxonomic (B) and function (C) composition of the fecal microbiome of cats (*n* = 16) fed either a retorted (circle) or extruded (triangle) diet. Samples of the same color and shape indicate samples from the same diet and year. From Bermingham et al 2018 and unpublished results. These graphs show that a) cats fed the retorted diet had higher microbial diversity compared to the cats fed the extruded diet irrespective of age, b) that young cats (triangle and circle) had a distinct microbial profile (i.e., they cluster differently) compared to older cats; this figure also shows that the cats fed the retorted diets had a different microbial population than cats fed the extruded diet and c) that the function of the microbiome is different between cats fed the extruded (triangle) and retorted (circle) diets; there is less separation associated with age with the exception of the young cats fed the extruded diets (pink triangle).

Microbial changes to the gastrointestinal tract in older dogs are characterized by a decrease in microbial diversity, potentially changing the way that older dogs can respond to diseases, regulate nutrient absorption, and energy and protein metabolic efficiency by peripheral tissues (Mizukami et al., 2019; Ghosh et al., 2022; Suchodolski, 2022). In the cat, microbial diversity has been observed to be relatively consistent with age (Figure 3a). However, both taxonomic composition differs with age (Figure 3b and 3C, respectively) with changes observed dependent on the diet consumed. Given that numerous bacterial species are known (in omnivore models at least) to alter gut permeability (Ulluwishewa et al., 2015), and that gut microbiota composition (Bermingham et al., 2018) and its metabolic potential (Deusch et al., 2015) changes with age, it is likely that the microbiota play a critical role in health and wellbeing during ageing and is certainly an area of importance for our understanding ageing in the pet.

### Age-associated conditions in cats and dogs

The mechanisms of ageing in the cat and dog have recently been reviewed (McKenzie, 2022). They identify that ageing is a complex inter-related process, which includes inflammageing, sarcopenia, insulin resistance, obesity, and cellular senescence (McKenzie, 2022). These processes accumulate to increase frailty, decrease tissue function, and increase disease risk and mortality. Research gaps associated with the mechanisms of ageing specifically in the cat and dog are indicated in Table 2. Interesting, despite inflammageing being touted as an underpinning condition for ageing cats and dogs, there is limited information in the two species to support the role of inflammageing associated with ageing diseases including sarcopenia and cognitive decline. Indeed, the authors concluded their comprehensive review by saying ‘a great deal of additional research is needed to clarify the details of ageing mechanisms [in dogs and] cats’. Indeed, recent approaches such as the MARS PETCARE BIOBANK™ (Alexander et al., 2023), “Generation Pup” (Murray et al., 2021) and the Ageing Dog Project (Bray et al., 2023) may one day clarify these mechanisms.

**Table 2.** Research Gaps associated with ageing mechanisms in the cat and dog summarized from McKenzie (2022)

Area	Mechanism	Gap
Cellular and molecular ageing	DNA damage	No information pertaining to nutritional modulation of DNA damage in ageing cats and dogs
	Epigenetic clock	Little evidence understanding how epigenetic clocks represent biological ageing in cats
	Intercellular signalling	Limited information around inflammageing in the cat and dog and nutritional improvement of this process
Tissue	Sarcopenia/muscle	No evidence to support elevation of cytokines (inflammageing) associated with sarcopenia in ageing dogs
		Limited evidence to support role of inflammageing in sarcopenia in ageing cats
		No information pertaining to role of insulin in ageing of feline muscle cells
		Limited evidence to support function of satellite muscle cells in ageing cats and dogs
		No evidence to support loss of type II muscle cells in ageing cats
		No evidence to understanding the role of exercise interventions on longevity in the cat or dog
	Bone	Limited evidence to support changes in bone structure in ageing cats
		Limited evidence to support the role of bone marrow stem cells in bone ageing in dogs
		Limited evidence to support the role of Growth Hormone and Interleukin Growth Factor-1 (IGF-1) in the ageing cat
		No information pertaining to the role of inflammation in the loss of bone associated with age in the cat and dog
		No information pertaining to the role of insulin in age-related loss of bone in the cat and dog
		No information pertaining to the role of exercise in maintaining bone health and function in the ageing cat and dog
	Joint Disease	No information pertaining to the role of chronic inflammation on development of joint disease in the ageing cat
		No information pertaining to the role of exercise in preventing Osteoarthritis (OA) in the ageing cat and dog
	Brain	No information pertaining to the role on chronic inflammation on the ageing brain is unknown in the cat and dog
		No information pertaining to the role of cellular senescence in the ageing brain in the dog
		No information pertaining to the role of physical activity on the ageing brain in the cat and dog
		Specific ageing processes unknown in the ageing cat brain (e.g., cellular senescence, inflammageing)
	Adipose tissue	Limited evidence to support the role of adipose mass, distribution and function in the ageing cat
		Limited evidence to support the development of sarcopenic obesity in the ageing cat and dog
		Limited evidence to support the role of inflammageing in fat mass distribution and function in the cat and dog
		No information pertaining to the role of exercise on fat mass distribution and function in the ageing cat and dog

Table 3 outlines observations from longitudinal studies published in the cat and dog. Earlier studies, tended to focus on outcomes related to lean body mass and diet, whereas more recent studies have assessed inflammageing (i.e., inflammatory markers) and changes to the fecal microbiome as a proxy for the gastrointestinal microbiome. In the cat, there are few studies assessing life span changes, but preliminary evidence suggests that diet appears to affect the way in which cats age (Table 3). A study using data obtained from veterinary visits from approximately 2 million cats and 4.4 million dogs, identified that the incidence of disease and age were interlinked (Salt et al., 2023). For example, younger cats were more likely to be ‘healthy’ or present for conditions such as fleas or parasite infections, whereas older cats were more likely to present with conditions such as ‘underweight’, periodontal disease, arthritis and renal failure. While trends in the dog were somewhat affected by body size (i.e., toy vs large breeds), typically younger dogs were classified as ‘healthy’ whereas older dogs typically presented with conditions such as ‘underweight’, ‘geriatric pet’ arthritis/osteoarthritis, heart failure, renal failure. Interestingly, these conditions appeared to occur at earlier chronological age in medium and large dog breeds. It is the hope of these authors that this data can be eventually used to target dietary interventions to improve the healthspan of our cats and dogs.

**Table 3.** Outcomes of longitudinal studies in healthy cats and dogs

Species	Age at beginning of study; mean (range)	Length of study	Sample size	Breed	Diet	Observation	Reference
Cat	7–17 years; groups: 7–9, 10–12, 13 + years	7 years	90; 30 per age group	Mixed breed	Extruded diet with/without antioxidants, a prebiotic, and omega-3 and omega-6 fatty acids	Cats fed diet with nutritional additives had increased lifespan of one year, greater serum vitamin E, HCT, haemoglobin, and RBC count, slower loss of BW, lean body mass, and skin thickness with age, and a tendency to be more active	Cupp et al. (2008), Cupp and Kerr (2010)
Cat	8 weeks	11 years	21; 10–11 per group	Domestic shorthair	Extruded vs. retorted diet	ATTD, fecal microbiome (composition, function), plasma inflammatory markers, plasma lipidome and metabolome were affected by age and diet	Bermingham et al. (2018) and unpublished data
Dog	1.9–8.1 years	13 years; adulthood to end of life	80	Labrador retriever	Extruded diet with/without avocado extract (< 0.10 %)	Age related increase in inflammation, oxidative stress, and tissue damage similar to “inflammageing” in humans with diet having no effect	Alexander et al. (2018)
Dog	5.5 years (4.3–7.5)	Median 14 years; puppy to end of life	39	Labrador retriever	Extruded diet	Lean and fat mass may influence longevity in the dog	Penell et al. (2019)
Dog	8 weeks	Median 13 years; puppy to end of life	48	Labrador retriever	Extruded diet with/without 25 % caloric restriction	Caloric restricted dogs lived longer and had delayed signs of chronic disease with no effect on skeletal structure/function	Lawler et al. (2008)
Dog	8 weeks	14 years; puppy to end of life	48	Labrador retriever	Extruded diet with/without 25 % caloric restriction	Caloric restricted dogs had healthier hips; reduced occurrence and delayed onset of OA	Smith et al. (2006)
Dog	8 weeks	8 years	48	Labrador retriever	Extruded diet with/without 25 % caloric restriction	Caloric restricted dogs had less severe OA	Kealy et al. (2000)
Dog	0.2–5.9 years	~ 8 years	6084	Labrador retriever	N/A—observational cohort in homes	Gastrointestinal illnesses in dogs may be affected by, location, presence of other animals in the home, and characteristics of owners	(Pugh et al., 2017)
Dog	0.7–5.0 years	~5 years	~6,000	Labrador retriever	N/A—observational cohort in homes	Genetics may play a role in limber tail in addition to being a working dog in a higher latitude	Pugh et al. (2016)
Dog	≥ 8 weeks and < 2 years	N/A	2,764	Golden retriever	N/A—observational cohort in homes	Dogs who have undergone gonadectomy had increased risk of becoming overweight/obese and dogs who were gonadectomised before 6 months of age had increased risk of orthopaedic injury compared to intact dogs	Simpson et al. (2019)
Dog	8 weeks—2 years	~1 year	160	Labrador retrievers, golden retrievers, and Labrador × golden crosses	N/A—observational cohort in homes	Cognition at young adult life stage can be predicted from early development stages	Bray et al. (2021)

Abbreviations: HCT, haematocrit; RBC, red blood cell; BW, body weight; ATTD, apparent total-tract digestibility; OA, osteoarthritis.

## Nutrition for the ageing cat and dog

There are a number of excellent resources for veterinary practitioners that consider the nutritional management of senior cats and dogs, for example the American Animal Hospital Association (AAHA; Dhaliwal et al., 2023) or the American Association of Feline Practitioners (AAFP; Ray et al., 2021). However, from a pet food regulatory point of view, while the NRC 2006, AAFCO and FEDIAF outline nutritional guidelines for gestation/lactation/growth and adult life stages, none include specific requirements for senior pets, even at the macronutrient (i.e., protein and fat) level. FEDIAF released a statement of the nutrition of senior dogs in 2017 (FEDIAF, 2017), which called for the industry to pay particular attention to specific nutrients such as crude fiber (to maintain gut motility) vitamin E, zinc, selenium, and docosahexaenoic acid (DHA); however, it was unable to provide specific recommendations around the minimum requirements for senior dogs due to “the lack of information available”. Additionally, it stated that the industry should work within the maximal levels of any particular nutrient stated by FEDIAF/AAFCO; this is especially important with nutrients such as eicosapentaenoic acid (EPA) and DHA which have safe upper limits identified for various health concerns. Finally, many of the ingredients which have been shown to have beneficial effects on the ageing pet (Table 4) have no labelling requirements—i.e., minimum or suggested inclusion levels are not regulated, making it difficult for pet owners to interpret whether ‘senior’ diets really provide additional benefits for their ageing pets. Indeed, recent research identified no nutritional differences (with the exception of crude fiber) in diets marketed for ‘adults’ or ‘seniors’ (Summers et al., 2020). However, it is apparent that nutrition, including water intake, can ameliorate the health conditions associated with age, or the underlying physiological process (Table 4). This suggests that current nutritional guidelines, including energy requirements which underpin feeding guidelines need dedicated scientific investigation to determine the nutritional needs for our senior and older cats and dogs.

**Table 4.** Nutrients with proven efficacy in cats and dogs.

Species	Age (range)	Intervention Target	Nutrient	Marker	Reference
Dog	1.7–10.6 years	Immune status	β-Carotene (20 or 40 mg β -carotene/kg diet)	Improved immune response	(Massimino et al., 2003)
Dog	7–10 years	Immune status	α-Tocopherol acetate/vitamin E (101 mg/kg diet)	Improved immune response	(Hall et al., 2003)
Dog	1.7–5.4 years	Immune status	Omega-6:omega-3 diet of 5:1 (dose not stated)	Positive effect on immune response	(Kearns et al., 1999)
Dog	1–4 years	Immune status	EPA (1.75 g/kg diet), DHA (2.2 g/kg diet) [omega-6:omega-3 of 3.4:1], sunflower oil (0.6 g/kg diet), menhaden fish oil (7 g/kg at 1.65% oil DMB)	Anti-inflammatory effects	(LeBlanc et al., 2008)
Cat	1.5–10 years	Immune status	Vitamin E (225 mg/kg DM diet)	Improved immune function	(O’Brien et al., 2015)
Cat	2.0–11.0 years	Immune status	Omega-6:omega-3 of 4.77:1 using salmon oil (dose not stated)	Improved immune system	(Rutherfurd-Markwick et al., 2013)
Dog	3 years	Cognitive function (behavior)	*Punica granatum* (457 mg/kg diet), *Valeriana officinalis* (260 mg/kg diet), *Rosmarinus officinalis* (0.44 mg/kg diet), *Tilia* species (635 mg/kg diet), *Crataegus oxyacantha* (392 mg/kg diet), _L_-Theanine (310 mg/kg diet), _L_-Tryptophan (329 mg/kg diet)	Improved neuroendocrine parameters associated with behavioral disorders (e.g., stress, anxiety, aggression)	Sechi et al. (2017)
Dog	2–12.6 years	Cognitive function	_D,L_-α-Tocopherol (1000 ppm), _L_-carnitine (250 ppm), _D,L_- α-LA (120 ppm), ascorbic acid 80 ppm, 1% inclusions of spinach flakes, tomato pomace, grape pomace, carrot granules and citrus pulp	Reduced cognitive dysfunction	Milgram et al. (2004)
Dog	2–12.5 years	Cognitive function	_D,L_-α-Tocopherol (1,050 ppm), _L_-carnitine (260 ppm), _D,L_-α-LA (128 ppm), ascorbic acid (80 ppm), 1% inclusions of spinach flakes, tomato pomace, grape pomace, carrot granules and citrus pulp	Reduced cognitive dysfunction	Milgram et al. (2002)
Dog	7–9 years	Cognitive function	α-LA (11.0 mg/kg diet), acetyl-_L_-carnitine (27.5 mg/kg diet)	Improved cognitive function demonstrated on two landmark discrimination tasks	Milgram et al. (2007)
Dogs	9–11.5 years	Cognitive function	Vitamin E (551 mg/kg diet), vitamin C (84.7 mg/kg diet), arginine (2.52 % as fed), thiamine (18.67 mg/kg diet), riboflavin (13.35 mg/kg diet), pantothenic acid (34.07 mg/kg diet), niacin (102.57 mg/kg diet), pyridoxine (11.05 mg/kg diet), cyanocobalamin (0.1 mg/kg diet), folic acid (3.94 mg/kg diet), EPA (0.24 % as fed), and DHA (0.21 % as fed)	Improved cognitive function (e.g., improved discrimination learning tasks)	Pan et al. (2018a)
Dogs	9–16 years	Cognitive function	6.5% MCT + Brain Protection Blend Vitamin E (552 mg/kg diet), vitamin C (151 mg/kg diet) arginine (1.79 % as fed), thiamine (58.7 mg/kg diet), riboflavin (26.5 mg/kg diet), pantothenic acid (77.3 mg/kg diet), niacin (225.76 mg/kg diet), pyridoxine (17.8 mg/kg diet), cyanocobalamin (0.175 mg/kg diet), folic acid (8.39 mg/kg diet), EPA (0.30 % as fed), selenium (0.681 mg/ kg diet), and DHA (0.23 % as fed)	Improved cognition scores (e.g., Senior Canine Behavior Questionnaire and a Canine Medical Health Questionnaire)	Pan et al. (2018b)
Dogs	6.8–8 years	Cognitive function	α-LA (30 mg (3 mg/kg BW)) and carnitine (60 mg (6 mg/kg BW))	Improved cognition (e.g., delayed recall aspect of delayed non-match to position (measuring short-term spatial memory) task)	Snigdha et al. (2016)
Dog	8–12 years	Cognitive Function	Vitamin E (800 IU or 210 mg/day (21 mg/kg BW/day)), vitamin C (16 mg/day (1.6 mg/kg BW/day)), carnitine (52 mg/day (5.2 mg/kg BW/day)), LA (26 mg/day (2.6 mg/kg BW/day))	Maintained cognition and reduced oxidative damage and Aβ pathology	Dowling and Head (2012)
Cat	5.5–8.7 years	Cognitive function	Vitamin E (550 mg/kg diet), vitamin C (80 mg/kg diet), arginine (2.3 % as fed), thiamine (55.0 mg/kg diet), riboflavin (30.9 mg/kg diet), pantothenic acid (55.4 mg/kg diet), pyridoxine (18 mg/kg diet), cyanocobalamin (0.09 mg/kg diet), folic acid (4.25 mg/kg diet), EPA (0.28 % as fed), and DHA (0.27 % as fed)	Improved cognitive function (e.g., egocentric learning, spatial memory)	Pan et al. (2013)
Cats	7–17 years	Longevity	Vitamin E (140.7 IU/1000 kcal), ß-carotene (5 mg/1000 kcal)	Increased longevity, and reduced disease incidence	Cupp et al. (2007)
Cats	7–17 years	Longevity	Vitamin E (140.7 IU/1000 kcal), ß-carotene (5 mg/1000 kcal), linoleic acid (21.3 % of dietary fat), chicory root (dose not stated)	Increased longevity, reduced disease incidence and improved intestinal health	Cupp et al. (2007)

Abbreviations: OA, osteoarthritis; BW, body weight; HA, hyaluronic acid; EPA, eicosapentaenoic acid; DHA, docosahexaenoic acid; ETA, eicosatetraenoic acid; DMB, dry matter basis; DM, dry matter; LA, lipoic acid; MCT, medium chain triglycerides.

There are a number of health concerns affecting senior pets, including hydration status and oral health (Salt et al., 2023), this review will summarize literature pertaining to sarcopenia, inflammation/inflammageing, and cognitive health.

### Sarcopenia

Maintenance of lean body mass is a major predictor of lifespan in both the cat and dog, having the most profound impact on mobility. Sarcopenia is defined as the loss of skeletal muscle mass and function with ageing. Paradoxically, this loss of muscle mass often occurs in the presence of obesity, so-called sarcopenic obesity (Freeman, 2012; LaFlamme, 2016). Approximately 40% of ‘old’ cats and dogs (Mao et al., 2013) are obese, and 12% to 15% of these are considered to have extremely low levels of lean mass. Therefore, this is a very common condition in older companion animals, although its mechanism are not well understood (McKenzie, 2022). Sarcopenia results primarily from impaired protein synthesis, with a smaller contribution from increased protein degradation, which ultimately leads to atrophy of skeletal muscle fibers and reduced mitochondrial function (Greenlund and Nair, 2003). A long-term study showed that in the elderly cat, fortification of a high protein and high fat diet with linoleic acid reduced the loss of lean body mass (Cupp et al., 2007).

### Inflammageing

Ageing is associated with a natural deterioration in health; more recently, the term inflammageing is used. This is the age-associated decline in immune function that occurs in most species. It is generally thought that inflammageing is influenced by the interactions between the host’s immune response and gastrointestinal microbiome (Fransen et al., 2017; Franceschi et al., 2018), but this has not been investigated in the cat and dog directly, nor are the cellular mechanisms well defined in the cat and dog (McKenzie, 2022). Inflammageing is believed to be the leading cause of morbidity and mortality in cats and dogs (Day, 2010); interestingly, many of the health conditions associated with ageing identified by Salt et al. (2023) such as osteoarthritis, renal failure and heart failure are underpinned by changes in inflammatory markers in both the cat and dog. Indeed, pro-inflammatory cytokines increase while anti-inflammatory cytokines decrease in the cat around the age of 8-10 years (Kipar et al., 2005). Nutrients such as β-carotene (a pre-cursor of vitamin A for dogs), vitamin E (tocopherol), and poly-unsaturated fatty acids (PUFA; typically supplied via fish/flax seed oil or algae) have been observed to improve immune status in cats and dogs (Table 4).

### Cognitive function

Dogs, and to a lesser extent cats, have often been used as a model for cognitive decline in humans; from this perspective there are a relatively large number of studies that have investigated the impacts of dietary nutrients on markers of cognition, including stress and anxiety. Nutrients, typically in proprietary blends, such as dietary lipids (PUFA, medium-chain triglycerides, and phospholipids), antioxidants, B-vitamins, carnitine, and specific amino acids such as arginine have been observed to improve markers of cognitive function in the ageing dog and to a lesser extent, the cat (Table 4).

## Conclusion

Our pet cats and dogs are living longer lives. As a consequence, more specific research into energy, macronutrient (protein, fat) and micronutrient requirements of our senior + pet cats and dog is required. Given that many of the conditions associated with ageing, or the underpinning physiological processes such as the immune system, respond to nutritional interventions in experimental settings suggests that ageing cats and dogs *do* have specific nutrient requirements. Therefore, research to better understand the energy and nutritional requirements is necessary and will hopefully enable the development of specific nutritional guidelines for our ageing cats and dogs.

## About the Authors

**Dr. Emma Bermingham** obtained her PhD in Animal Science at Massey University in New Zealand and went on to do a number of post-doc positions in Australia, France, and the UK. She has 18+ years experience in pet nutrition, including at FMCG roles, and fundamental research programs focusing on food–microbe–host relationships in both the cat and dog. She has a special interest in understanding the impacts of high dietary protein on the composition and function of the microbiome. More recently, she has been researching the role of diet, intestinal function, and age in the domestic cat. **Corresponding author:**emma.bermingham@gmail.com

**Keely Patterson** is a PhD student at Massey University in New Zealand and sponsored by AgResearch, where she is a part of the Food Chemistry and Structure Team. She completed her Bachelor Science honors degree in animal biology with a minor in nutrition and nutraceutical sciences from the University of Guelph, Canada and worked in Dr. Shoveller’s lab upon completion. Keely’s current research focuses on the gut health of ageing cats, specifically using a novel method to measure intestinal permeability and absorptive capacity using liquid chromatography-mass spectrometry.

**Dr. Anna Kate Shoveller** received her BSc in Animal Biology from the University of Alberta and a PhD in Nutrition and Metabolism from the University of Alberta. She is currently a professor in the Department of Animal Biosciences at the University of Guelph. Previously, she was employed by Procter & Gamble and Mars Pet Care where she added to the knowledge of dog and cat nutrition through investigation in the areas of energy metabolism and nutrient budgets of dogs and cats using indirect calorimetry and applying isotope dilution methodologies to quantify amino acids requirements in adult dogs. She has taken this experience and returned to academia where she teaches Companion and Equine nutrition at the undergraduate level and Comparative Nutrition at the graduate level. She also runs an active comparative nutrition research group where her research focuses on amino acid, fatty acid and energy metabolism in dogs, cats, and horses.

**Dr. Karl Fraser** is a Senior Research Scientist, Nutritional Metabolomics, Food Chemistry and Structure Team in the Food Bio-based Products Group at AgResearch, New Zealand. He obtained his PhD from the University of Auckland, New Zealand in Chemistry and focused on the development and application of mass spectrometry to the metabolomics of food. He has worked on a diverse range of projects, ranging from those related to forage-based agricultural systems, i.e., forage, meat, and milk metabolomics, to more recently biomarker discovery in the area of human health and nutrition.

**Dr. Nina Butowski** obtained her Master’s degree in Veterinary Bioscience at the University of Glasgow, which included a year’s research as an industrial placement student at the Waltham Petcare Science Institute, Mars Petcare. She went on to obtain her PhD in Animal Science from Massey University, New Zealand. Nina is currently a Scientist based at AgResearch Ltd, New Zealand. Her research focuses on the effect of diet on the host gastrointestinal microbiome, and how this can be modulated. She employs an omics approach to investigate the microbes present, their metabolites, and interactions with the host.

**Dr. David Thomas** obtained his Bachelor of Science honours degree in Zoology at the University of Dundee, before obtaining his PhD in Zoology at University College London. He has been employed at HortResearch Ltd in New Zealand and is currently an associate professor at Massey University, New Zealand. His main research focus is on seasonality in companion animals and the implications of this on nutritional requirements and nutritional approaches to maintain health in geriatric cats and dogs.
